# Tumor antigens as proteogenomic biomarkers in invasive ductal carcinomas

**DOI:** 10.1186/1755-8794-7-S3-S2

**Published:** 2014-12-08

**Authors:** Lars Rønn Olsen, Benito Campos, Ole Winther, Dennis C Sgroi, Barry L Karger, Vladimir Brusic

**Affiliations:** 1 Bioinformatics Centre, Department of Biology, University of Copenhagen, Copenhagen, Denmark; 2 Biotech Research and Innovation Center (BRIC), University of Copenhagen, Copenhagen, Denmark; 3 Cancer Vaccine Center, Dana-Farber Cancer Institute, Harvard Medical School, Boston, MA, USA; 4 Division of Experimental Neurosurgery, Department of Neurosurgery, Heidelberg University Hospital, Heidelberg, Germany; 5 Cognitive Systems, DTU Compute, Technical University of Denmark, Lyngby, Denmark; 6 Massachusetts General Hospital, Harvard Medical School, Boston, MA, USA; 7 Barnett Institute, Northeastern University, Boston, MA, USA 02115; 8 Department of Medicine, Harvard Medical School, Boston, MA, USA; 9 Department of Computer Science, Metropolitan College, Boston University, Boston MA, USA; 10 School of Science and Technology, Nazarbayev University, Astana, Kazakhstan

**Keywords:** Proteogenomics, biomarker, cancer, tumor antigens, protein-protein interaction, protein expression, gene expression

## Abstract

**Background:**

The majority of genetic biomarkers for human cancers are defined by statistical screening of high-throughput genomics data. While a large number of genetic biomarkers have been proposed for diagnostic and prognostic applications, only a small number have been applied in the clinic. Similarly, the use of proteomics methods for the discovery of cancer biomarkers is increasing. The emerging field of proteogenomics seeks to enrich the value of genomics and proteomics approaches by studying the intersection of genomics and proteomics data. This task is challenging due to the complex nature of transcriptional and translation regulatory mechanisms and the disparities between genomic and proteomic data from the same samples. In this study, we have examined tumor antigens as potential biomarkers for breast cancer using genomics and proteomics data from previously reported laser capture microdissected ER+ tumor samples.

**Results:**

We applied proteogenomic analyses to study the genetic aberrations of 32 tumor antigens determined in the proteomic data. We found that tumor antigens that are aberrantly expressed at the genetic level and expressed at the protein level, are likely involved in perturbing pathways directly linked to the hallmarks of cancer. The results found by proteogenomic analysis of the 32 tumor antigens studied here, capture largely the same pathway irregularities as those elucidated from large-scale screening of genomics analyses, where several thousands of genes are often found to be perturbed.

**Conclusion:**

Tumor antigens are a group of proteins recognized by the cells of the immune system. Specifically, they are recognized in tumor cells where they are present in larger than usual amounts, or are physiochemically altered to a degree at which they no longer resemble native human proteins. This proteogenomic analysis of 32 tumor antigens suggests that tumor antigens have the potential to be highly specific biomarkers for different cancers.

## Background

Cancer cells differ from normal cells by genetic and epigenetic aberrations in a number of cellular functions. The genetic hallmarks of cancer include self-sufficiency in growth signals, insensitivity to growth-inhibition signals, unlimited replicative potential, resistance to apoptosis, sustained angiogenesis, and local tissue invasion and metastasis [1]. In addition, cancer cells display deregulated cell energetics, instability and mutation of the genome, avoidance of immune destruction, and tumor-promoting inflammation [2]. Epigenetic changes include DNA methylation, histone modifications, nucleosome positioning, and miRNA expression [3]. Furthermore, carcinogenesis could be explained by interactions between cancer cells and surrounding tissues [4]. These hallmarks define functional profiles and aberrations that distinguish cancer from normal tissue.

Immune evasion employed by tumors involves multiple cellular and molecular mechanisms. Examples include blocking of the STAT-3 signaling pathway [5], toll-like receptor activation [6], production of immunosuppressive cytokines [7], infiltration of myeloid suppressor cells or regulatory T cells [8,9], activation of immunosuppressive networks [10], and down-regulation of human leukocyte antigen or impairment of antigen processing and presentation [11,12]. Inflammation promotes multiple hallmark capabilities through a spectrum of bioactive molecules that are supplied to the tumor microenvironment in support of the hallmarks of cancer [13-18]. These include growth factors that support proliferative signaling, and survival factors that reduce cell death. Inflammation also promotes the transformation of epithelial cells that enables cancer cells to invade, to resist apoptosis, and to spread to other tissues. Inflammation also promotes factors that stimulate angiogenesis, local tissue invasion, and metastasis.

Tumor antigens (TAs) are tumor proteins that, when expressed in tumors, are recognized by the host immune system. They represent markers that are either specific for individual tumors or are generally overexpressed in tumors as compared to normal tissues [19]. TAs can be neoantigens (tumor-specific antigens) that arise from mutation or RNA splicing. Neoantigens are expressed only by cancer cells and not by normal tissue [20]. Tumor-associated antigens show increase in expression in cancer tissue as compared to normal tissue (e.g. IDO1 [21], HER2 [22], or survivin [23]). Tissue-specific antigens that are recruited and expressed by cancers in specific tissues (e.g. cyclin-A1 [24] or Cancer/Testis Antigen 1B [25]). TAs are targets for cancer diagnostic and therapy that have been studied extensively - more than 1400 clinical trials focusing on TAs have been reported in clinicaltrials.gov as of October 2013 http://www.clinicaltrials.gov.

Most common biomarkers are based on patterns in expression of genes, RNA, proteins, or epigenetic patterns [26]. Examples of cancer biomarkers routinely used in the clinic include α-fetoprotein (AFP) for diagnostics and management of testicular cancer [27], *MUC16 *(cancer antigen 125 or CA-125) for ovarian cancer [28], *ERBB2 *(HER2) protein for breast cancer [29], and prostate specific antigen for prostate cancer [30].

The progression of a normal cell towards a neoplastic state comprises a cascade of events that are responsible for inducing tumorigenesis [31]. The conditions of the tumor microenvironment may also contribute to the cellular characteristics of tumor cells, as this microenvironment may induce genetic instability in tumor cells [32].

The advent of high-throughput genomics methods has facilitated a dramatic increase in the number of candidate genetic biomarkers [33]. However, very few new genetic biomarkers have been added to the clinical toolbox in recent years [34]. Approximately 95% of human protein coding genes produce splice variant transcripts increasing the number of potential gene expression biomarker candidates [35]. Individual genetic biomarkers are rarely informative as genetic redundancy provides for high genetic flexibility without necessarily affecting the biological phenotype [36]. The expression of many genes correlate with disease progression in individual patients, but the analyses of large data sets consistently show that only a small subset of these is consistently observed in larger cohorts. The expression of genes may also prove inconsistent over time, since cells respond to changes in environment and undergo transcriptomic changes in different stages of development. While transcriptomic differences are apparent between normal and cancerous tissues, the expression of TAs remains relatively constant through different cancer stages, suggesting that most defining genetic alterations conferring a cancerous potential occur at the early stages of tumorigenesis [37]. In contrast, similar studies of the tumor microenvironment reveal extensive gene expression changes in tumor stromal tissue during cancer progression [38].

The number of protein biomarkers has been growing, owing largely to the advances in mass spectrometry techniques [39]. However, protein biomarkers, similar to genetic biomarkers, are rarely uniquely expressed (or overexpressed) in malignant tissues [34]. At the same time, more than 260,000 protein variants resulting from alternative splicing have been annotated to date [40]. Additionally, a variety of post-translational modifications (PTMs) can alter the structure and function of proteins [41]. Most of the proposed protein biomarkers are not commonly used in clinical application as they do not hold up statistically in large populations, or the cost of assays outweighs their prognostic value [42]. Protein expression profiling has proven useful for stratification of cancer versus normal tissues for invasive ductal carcinoma. Differentially expressed proteins or protein sets can be used as candidate biomarkers for carcinogenesis [43].

Relative to the research effort, the number of verified useful biomarkers is vanishingly small [44] and we need new and improved approaches. The emerging field of proteogenomics seeks to synergistically enrich the value of genomics and proteomics by studying the intersection of the two data sets [45]. True integrative analysis of genomics and proteomics data is a non-trivial task, as the expression of mRNA typically does not always correlate well with the expression of corresponding proteins [46]. The mechanisms of transcriptional and translational regulation have been extensively studied, but are not yet fully understood. However, when the expression of individual genes or pathways correlate with protein expression, this may provide insights into transcriptional effects and the mechanistic basis of statistically derived biomarkers [47]. Proteogenomic profiling has been applied to the study of invasive ductal carcinomas (IDCs) to reveal potential biological events not previously associated with this cancer type. The study of biological networks of differentially expressed proteins and differentially expressed genes revealed patterns that correlate with clinical relapse [48].

In addition to their potential diagnostic and prognostic value as biomarkers, tumor associated antigens (TAAs) and tumor specific antigens (TSAs) have therapeutic potential as targets of cancer immunotherapies [49,50]. In this study, we have combined the analysis of protein and mRNA expression of a selection of well-described TAAs [51,52] to evaluate their proteogenomic potential as biomarkers for IDCs.

## Results and discussion

### Translation of mRNA in IDC tissue

The central dogma of molecular biology states that DNA is transcribed into RNA, which is in turn translated into protein. From this general rule, it is often assumed that a certain amount of DNA makes an equal, or at least proportional, amount of RNA, which in turn makes an equal or proportional amount of protein. However, it is increasing clear that a plethora of transcriptional and translational regulatory mechanisms affect the dynamics of the central dogma. Examples of transcriptional regulation include: miRNA interference [53]; epigenetic factors, such as methylation [54]; and alternative splicing [55]. Translational regulation affects the rates of degradation for different proteins [56], as well as PTMs [57]. Many of these regulatory mechanisms have been used in efforts to characterize new biomarkers for various cancers [58-61]. Although most of these mechanisms are not yet fully understood, they modulate transcription and translation in all cells.

The correlation of expression between 1066 gene/product pairs from 23 human cancer cell lines of various origins and cancer types was examined [62]. This study reported 169 genes for which mRNA and protein expression correlated (at threshold of ρ > 0.455). The study, involving the analysis of biological function ontologies, found that within the set of 169 genes, ontologies relating to the cytoskeleton and adherent junctions in the cellular compartment, cellular motility, and other maintenance-related categories were significantly enriched [62].

Dysregulation of cellular processes is a feature of cells undergoing tumorigenesis, and transcriptional and translational mechanisms are likely to be dysregulated as well. Given the heterogeneous nature of cancer, it is unlikely that transcription and translation modulation is homogenous in different cancers. This prompted us to examine the correlation between gene expression and protein expression in the Cancer Genome Atlas (TCGA) consortium IDC tissues, to provide an IDC specific context for our analysis of the unpaired sets of mRNA and protein expression data between normal, preinvasive and invasive breast cancer tissue.

**Figure 1:**
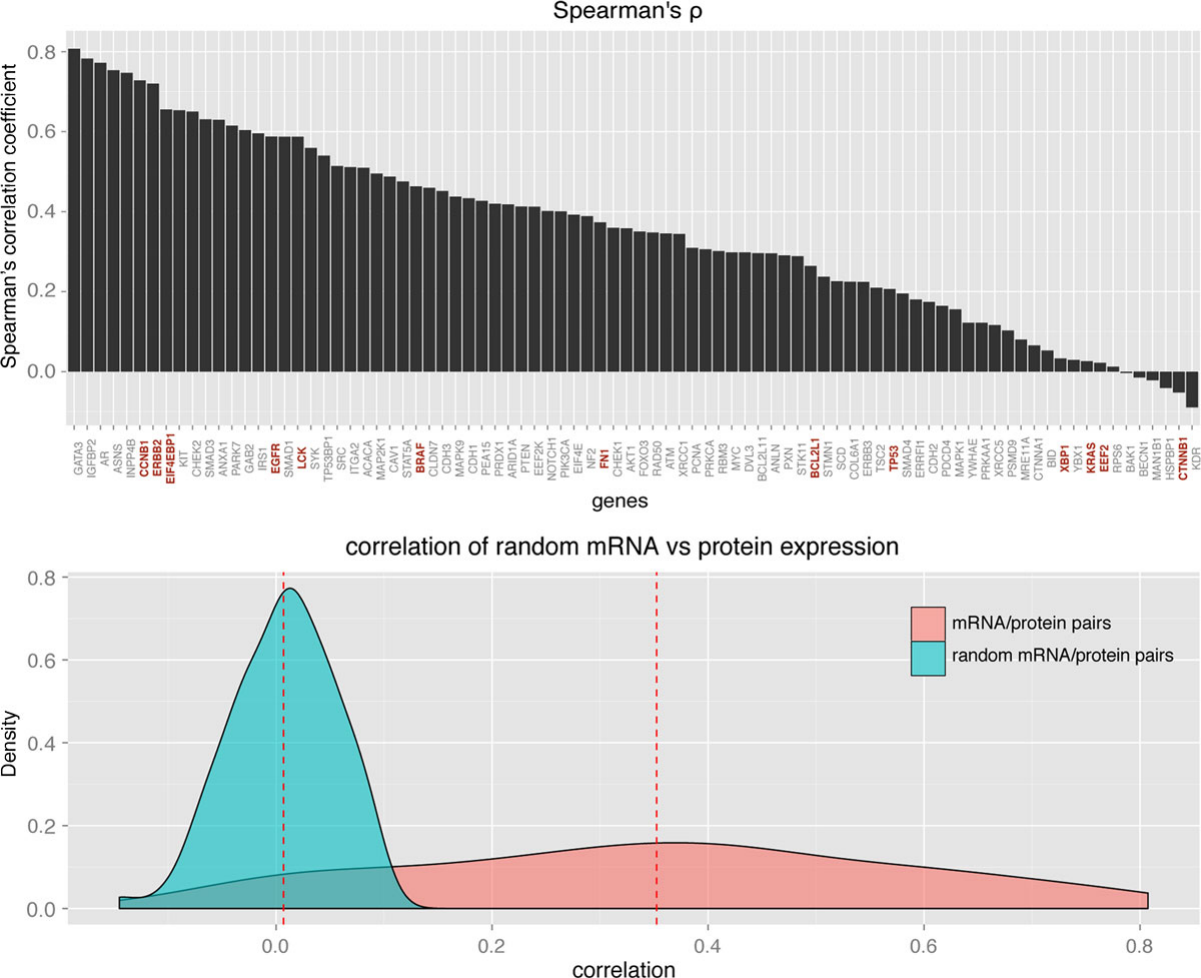
**Top panel: Spearman's rank correlation between mRNA expression and protein expression of 86 genes in 404 IDC patients**. The TAs are highlighted in red. Bottom panel: density distribution of correlation coefficients of mRNA vs protein expression (pink) and density distribution of correlation coefficients of randomized mRNA vs protein expression (aqua). The dashed red lines mark the mean correlation in each distribution.

Our results are consistent with the notion that it is insufficient to study only the expression of mRNA when searching for TAs as potential immunotherapy targets, but protein expression also needs to be considered. We calculated Spearman's ρ for the mRNA/protein expression pairs of the 86 genes examined by TCGA. Twenty-nine genes were found to have a ρ > 0.455, six of which are TAs (Figure 1, top panel). Although the number of examined proteins is too small to draw global conclusions (global patterns of translation in IDC tissue still remain to be elucidated in comprehensive analyses), we found that of 13 examined TAs, mRNA and protein expressions only correlate in six. This observation is consistent with the results reported in [62,63].

The mean correlation of expression of the 86 mRNA/protein IDC pairs was calculated to be 0.35. Randomly paired mRNA and protein expressions yield a mean correlation of approximately zero, indicating no apparent bias in the data. Distributions of correlation coefficients in the normal and randomized data set are shown in Figure 1 (bottom panel).

### Expression of tumor antigens on protein level

**Figure 2:**
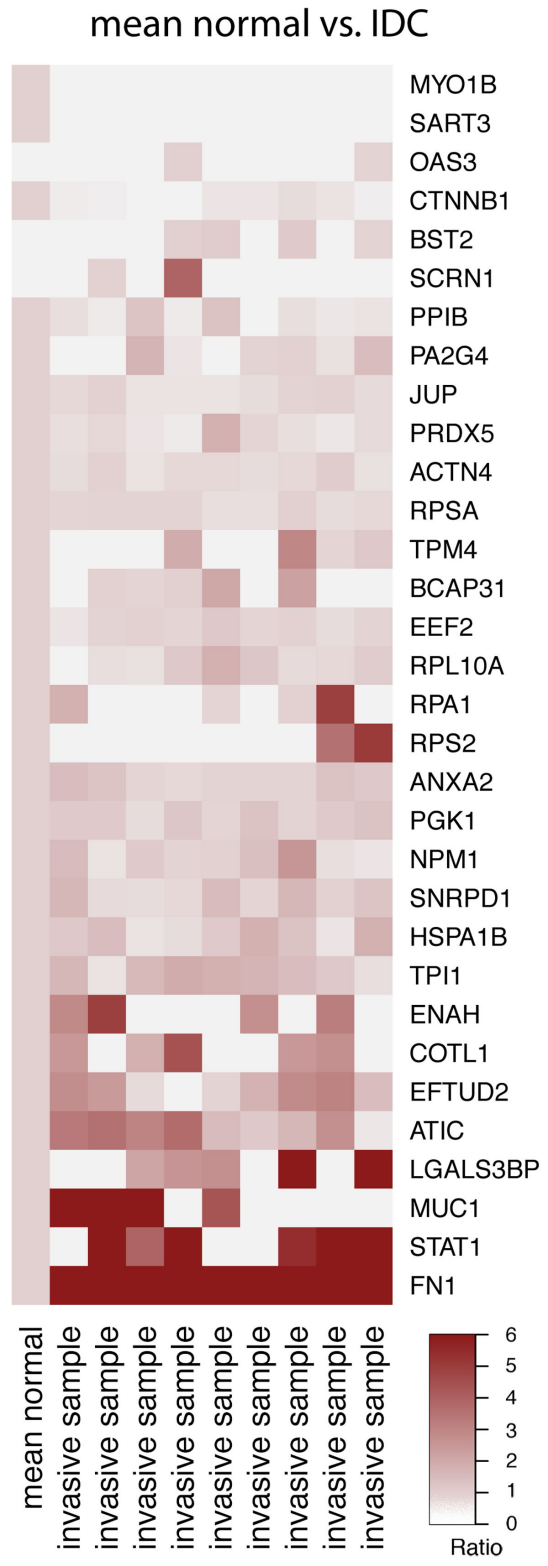
**Ratio of protein expression in IDC tissue to mean expression in normal tissue of 32 measured TAs**.

Although not paired like tissue samples used for the analysis of gene expression, the protein expression data offer valuable information for prescreening and filtering of TA genes for further analysis. With the exception of OAS3, BST2, and SCRN1, all TAs were expressed in at least one of the normal tissue samples [43]. Not all TAs were expressed in the IDC tissue, and only a fraction was consistently expressed in all nine patient samples (Figure 2). These results were not unexpected, since different expression patterns are often observed in different cells of the same tumor [64]. Within the small cohort examined here, 30 of 32 TAs were expressed as proteins in at least two of nine patients. The two TAs, MYO1B and SART3, found to not be expressed in the IDC patient cohort were removed from the list and the remaining 30 TAs were further analyzed.

### Tumor antigen gene expression patterns of normal, preinvasive and invasive IDC tissues

**Figure 3:**
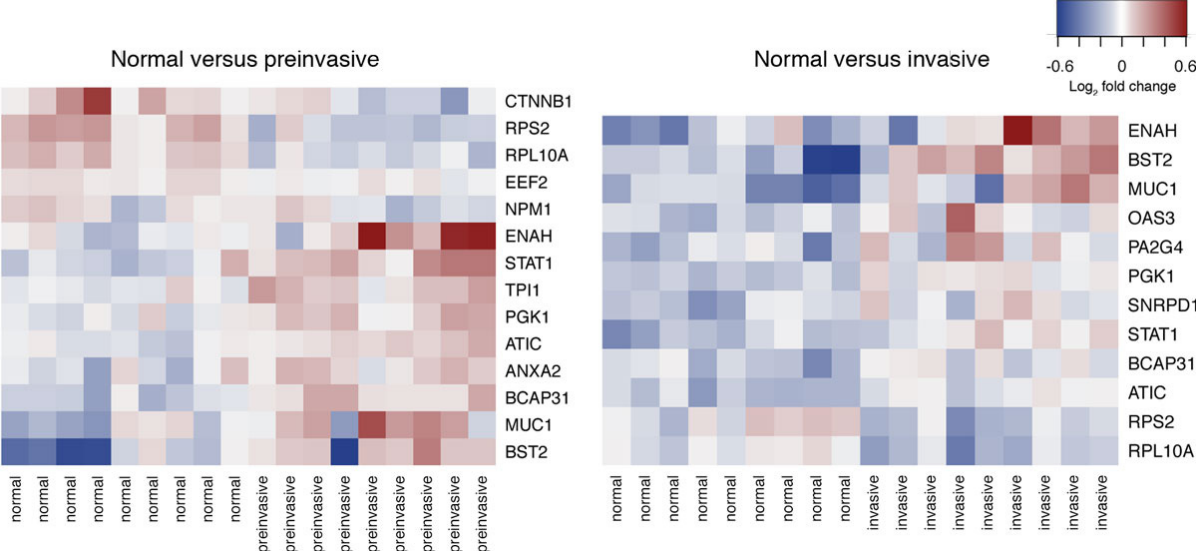
**The heat map based on log_2 _transformed fold change from median expression of TA mRNA**. Shown in these heat maps are TAs that are significantly differentially expressed between normal tissue and preinvasive tissue (left) and between normal tissue and invasive tissue (right) (p < 0.05). Red corresponds to up regulated and blue corresponds to down regulated transcripts.

We examined differential expression of TA mRNAs between normal and preinvasive IDC tissue for the TAs expressed as proteins in the IDC tissue. Of the 30 TAs expressed in IDC tissue (excluding MYO1B and SART3), mRNA expression data was measured for 28 TAs (not measured for RPSA and HSPA1B). Five TAs displayed consistent down regulation between the normal and preinvasive tissues, nine TAs displayed up regulation and 16 showed no significant difference between the two types of tissue (p < 0.05). Fold change to median expression of significantly differentially expressed TAs is shown in Figure 3 (left panel). A comparison of TA expression between normal and invasive tumor tissues revealed ten up regulated TAs and two down regulated TAs (Figure 3, right panel).

The cytoplasmic ribosomal subunits RPS2 and RPL10A were down regulated in both preinvasive and invasive tissues (Figure 3). These patterns, although not consistent with the increased protein synthesis by tumor cells, have been observed previously in breast cancer and colorectal cancer studies [38,65]. The gene expression of these TAs is typically up regulated in cancer, but it was down regulated in the IDC tissue. Sgroi and colleagues noted that the mechanisms by which ribosomal proteins contribute to tumorigenesis are relatively poorly understood [38] but it is known that changes in components of translational mechanism increase cancer risk [66]. The down regulation of these particular ribosomal subunits may be indicative of qualitative rearrangement of the ribosomal proteins as a whole, meaning that one should be careful in interpreting these observation points as potentially causal.

Seven TA genes were up regulated in both preinvasive and invasive tissues as compared to the normal tissue. The genes of the STAT protein family encode a series of signal transducers and transcription activators. *STAT1 *is up regulated in both preinvasive and invasive IDC tissues, as compared with the normal tissue. This pattern has been associated with the faster progression from ductal carcinoma *in situ *to invasive carcinoma, most likely by inducing immunosuppression in the tumor microenvironment [67]. *STAT1*, which is typically dormant in normal tissue, is also associated with aggressive growth and chemotherapy resistance [68]. Interestingly, there is very little difference between fold changes of *STAT1 *expression between preinvasive and invasive tissues compared with the normal samples, indicating that the effects of *STAT1 *are already present in the preinvasive tissue.

Likewise, the bone marrow stromal cell antigen 2 (BST2) is up regulated in the IDC. The up regulated *BST2 *gene has been proposed as a biomarker for bone metastasis of breast cancer [69]. Similar patterns have been observed in tamoxifen resistant breast cancer cells, where up regulation of *BST2 *gene expression correlated to increased invasiveness and metastasis, regulated and activated by *STAT3 *[70].

Increased expression of phosphoglycerate kinase 1 (PGK1) was also observed in both preinvasive and invasive tissues. PGK1 is a major enzyme in glycolysis and facilitates ATP production under hypoxic conditions [71]. The elevated levels of PGK1 have previously been associated with an increased invasiveness of gastric cancer [72]. PGK1 was reported to facilitate the release of the anti-angiogenic enzyme, angiostatin [73].

Mucin 1 (MUC1) is an oncoprotein that is overexpressed in 90% of breast cancer patients and its gene is amplified in 40% of the patients. Overexpression of this protein has been linked to tamoxifen resistance [74]. The activities of MUC1 leading to tamoxifen resistance are many: it contributes to the activation of the PI3K-AKT pathway known to be involved in apoptosis [75]. It activates the MEK/ERK pathway, a regulator of cellular growth and apoptosis in breast cancer [76]. It also activates the Wnt/β-catenin pathway involved in cell proliferation and migration as well as formation and maintenance of cancer stem cells [77], and it activates STAT pathways associated with cellular growth and inflammation [78].

We also found the cytoskeleton regulatory protein, ENAH, to be overexpressed in both preinvasive and invasive tissues. EHAH is non-detectable in normal tissues, but was found to be weakly expressed in the low risk benign lesions, and was overexpressed in the high-risk benign breast lesions [79].

ATIC, the product of the *purH *gene, is involved in the final steps of *de novo *synthesis of purine [80]. Imbalances in the biosynthesis and metabolism of purine is linked with progression of a number of cancer types [81]. ATIC inhibition has been explored as a therapeutic strategy in breast cancer patients [82], as has the potential of ATIC as a TA [83].

The function of B-cell receptor-associated protein 31 (BCAP31) is relatively unknown. It has been proposed to be involved in CASP8-mediated apoptosis, where its cleavage product is a strong inducer of apoptosis. However, caspase resistant types of BCAP31 have been observed and the lack of cleavage during apoptosis leads to reduced apoptotic potential [84]. BCAP31 has previously been reported to be up regulated in the breast cancer tissue as compared to normal tissue [85].

### Individual expression profiles

**Figure 4:**
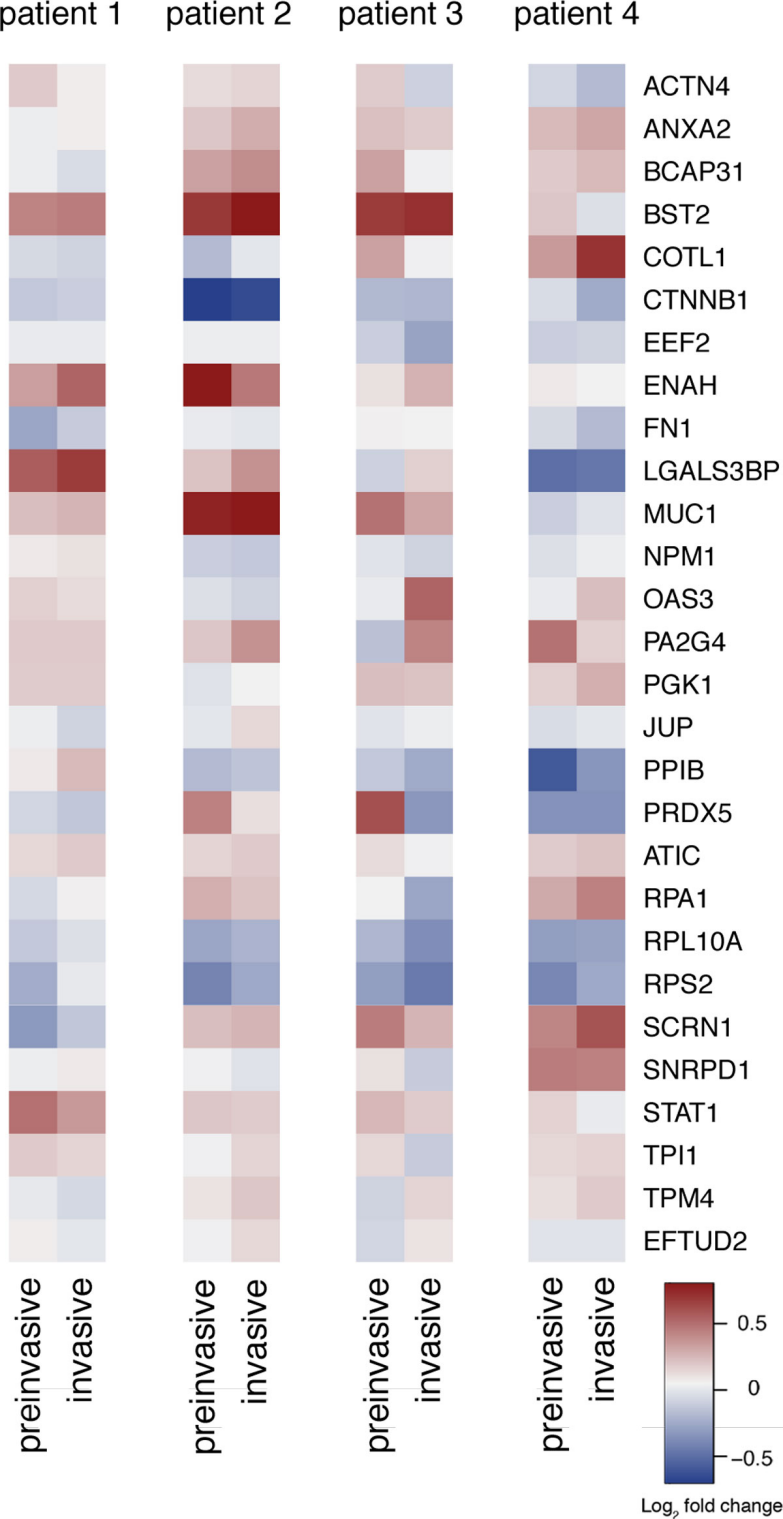
**Heat map based on log_2 _transformed fold change of gene expression in preinvasive and invasive IDC tissue compared with expression in normal tissue in four individual patients**.

Although there are similarities between TA genes differentially expressed in preinvasive and invasive tissues, there were also discrepancies between patients, even in this small cohort. The analysis of four patients for whom expression was measured in normal, preinvasive, and invasive tissues revealed that there are only a few clear patterns of expression (Figure 4). Only four TA genes, *ANXA2*, *ENAH*, *ATIC*, and *STAT1*, were consistently up regulated in both IDC tissue types in all four patients. Two TA genes, *CTNBB1 *and *RPL10*, were consistently down regulated in both IDC tissues in all four patients. No complex patterns, i.e. genes up regulated in one IDC tissue type and down regulated in the other, or vice versa, were observed. However, the expression of some TA genes were consistent across tissue types, but not across patients; for example, *LGALS3BP *is strongly expressed in patient 1, but down regulated in patient 4.

### Pathway-based assessment of expression patterns

To evaluate the potential biological impact of aberrantly expressed TAs, we examined their involvement in canonical pathways [86]. From the analyses of differential mRNA expression in the four individual expression profiles, it is clear that heterogeneity of mRNA expression exists even when examining a very limited number of proteins. Although only approximately half of the TA genes were significantly differentially expressed in the full cohort, all 30 TAs were aberrantly expressed in at least one of the four individual profiles, and all 30 were expressed at protein level. Of these 28 have entries in the STRING database.

**Figure 5:**
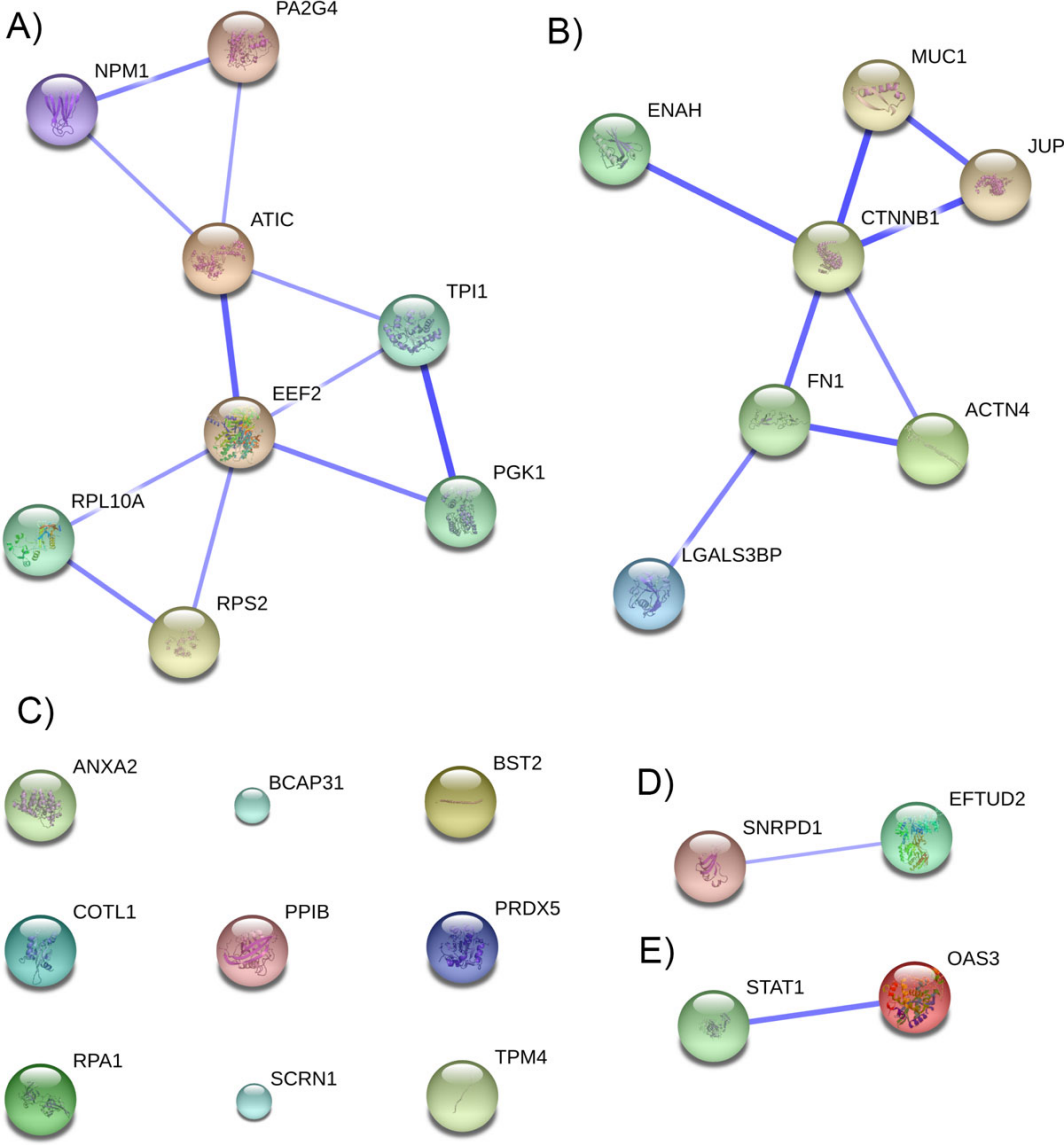
**Confidence view of protein-protein interactions within the 28 examined TAs, generated using STRING database**. Nodes correspond to TAs and edges correspond to functional interactions. Thicker edges signify higher confidence in the interaction. Only interactions with a confidence score higher than 0.5 were included.

The functional relationship between the 28 TAs was examined using the STRING database. An interaction confidence threshold of 0.5 yielded four functionally related groups as well as nine non-related TAs (Figure 5).

**Figure 6:**
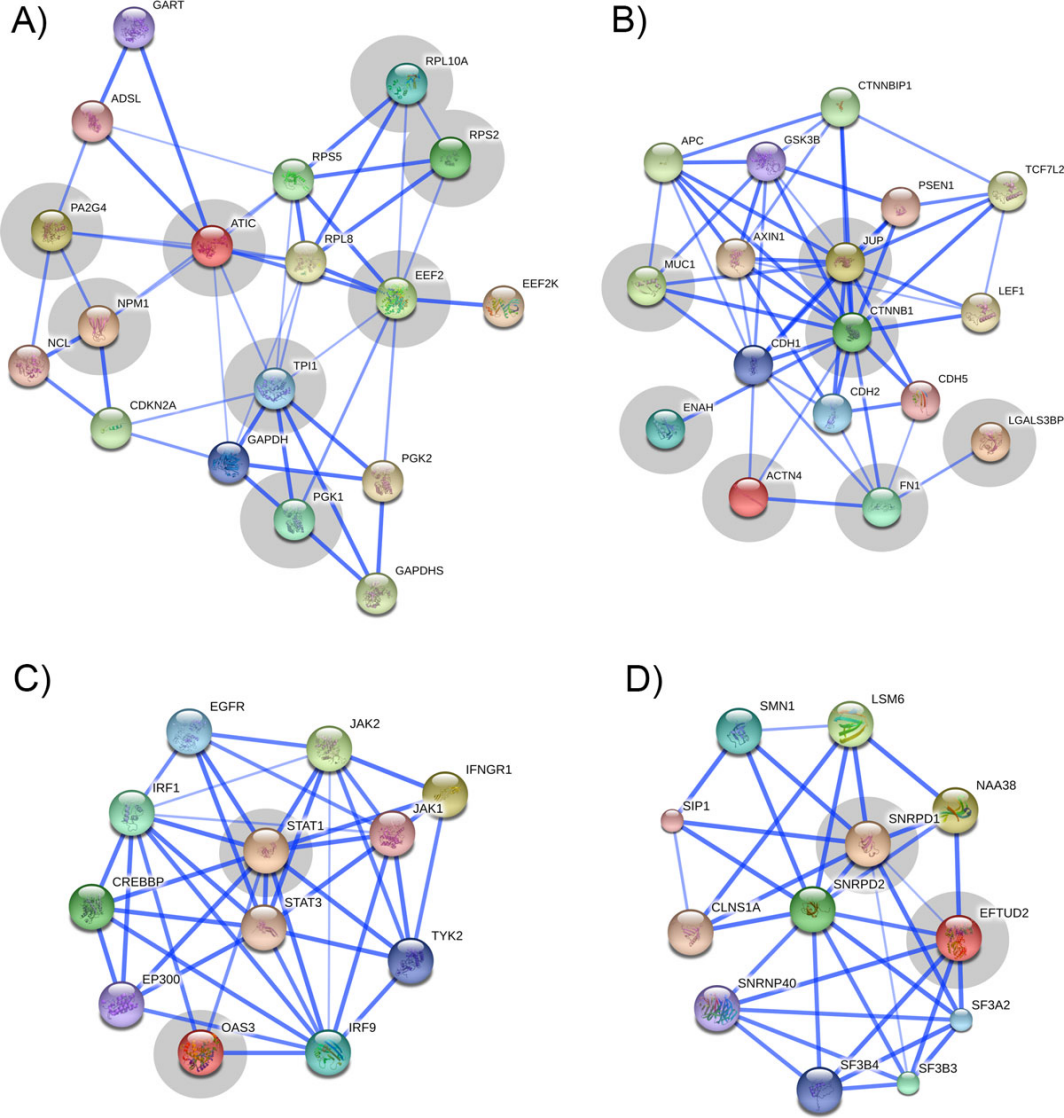
**Confidence view of expanded protein-protein interactions within the four functional groups of TAs (highlighted in gray) generated using STRING database**. Interacting proteins were added to the TAs using one cycle of expansion. Nodes correspond to the proteins and edges correspond to their functional interactions. The thicker edges signify higher confidence in the interaction. Interactions with a confidence score higher than 0.5 are shown.

The four functional groups were further examined for their involvement in the canonical pathways related to the hallmarks of cancer. One cycle of expansion to include related proteins was applied to each of the four groups to yield four functional modules (Figure 6). Further examination of each of the four functional modules and their direct interactants, using MSigDB [87], reveals dysregulation of canonical pathways related to the hallmarks of cancer.

#### Module 1

Expansion of group 1 (Figure 5A) yielded 10 direct functional neighbors at a confidence score of 0.5 (Figure 6A). The resulting PPI is heavily interconnected indicating tight functional homology within the module.

The proteins of functional module 1 overlap in two canonical pathways: PGK1, TPI1, GAPDH, and GAPDHS overlap in the glycolysis pathway, and expression of twelve of the eighteen proteins are known to be positively correlated with BRCA1 expression in BRCA1^mut ^tumors [88]. Both pathways have been extensively studied as potential therapeutic targets in cancer.

Aerobic glycolysis has profound effects on proliferating cells [89]. Abnormal cellular metabolism is a defining feature of cancer [1], and proteins involved in metabolic pathways have both diagnostic and therapeutic potential [90,91]. Numerous enzymes are involved in maintaining elevated rates of glycolysis, making them potential targets of therapeutic agents [92].

Mutations in the *BRCA1 *gene are associated with faster progression of breast cancer and other cancers [93]. BRCA1 plays a role in DNA repair and genomic stability maintenance. It is a known tumor suppressor, which, when mutated, is linked with the early onset of breast cancer. Network modeling strategies revolving around BRCA1^mut ^revealed a number of genes associated with centrosome dysfunction and thereby increased cancer aggressiveness [88]. Twelve genes in module 1 (six of these are TAs) were involved in the network functionally associated with *BRCA1 *mutations and centrosome dysfunctions.

#### Module 2

Module 2 consists of seven TAs (Figure 5B), which when expanded by one cycle in the STRING database has ten interaction partners in a highly connected PPI (Figure 6B). Seven proteins (CTNNB1, APC, GSK3B, AXIN1, LEF1, TCF7L2, PSEN1, CTNNBIP1) overlap in the Wnt pathway, and a subset of these additionally overlaps in the β-catenin pathway. A smaller subgroup consisting of TAs ACTN4, ENAH, and FN1 overlaps in an actin cytoskeleton regulation pathway. Both these pathways are generally regarded to play a role in cancer, and are known to be connected through shared regulators [94].

The Wnt pathway is involved in a number of cancer-relevant cellular processes such as cell division and migration, and cell fate decisions [95]. Elevated levels of β-catenin in the nucleus or cytoplasm of cells, indicates activation of the Wnt pathway, which correlates with poor prognosis in breast cancer patients [96], and detection of β-catenin levels using immunohistochemical staining is an important diagnostic tool [97]. Molecules of the Wnt pathway have been extensively targeted in anticancer treatment modalities, involving small interfering molecules, blocking antibodies, and peptide-based therapies [98].

#### Module 3

Module 3 (Figure 6C, generated by expansion of the TAs in Figure 5E) is centered on proteins relating to the immune response, as ten of the proteins in this module overlap in immune system related pathways. The predominant immune system pathways in this module are cytokine signaling (specifically IL-6), interferon signaling, and JAK/STAT signaling.

All three pathways have been associated with cancer development. The JAK/STAT pathway is recognized as a modulator of cytokine signaling, and has therefore been associated with a large number of malignancies [99]. In cancer, the JAKs and STATs (particularly STAT3) are often observed to be constitutively activated, which is thought to induce cell proliferation and prevent apoptosis [100]. Additionally, the STATs are known to induce pro-oncogenic inflammation in the tumor microenvironment by promoting pathways such as NF-κB and IL-6-GP130-JAK pathways [101]. A number of NF-κB encoded inflammatory factors, such as IL-6, are activators of STAT3, in turn creating a positive feedback loop, leads to dysregulated action of immune modulating pathways [102].

Another commonly observed immune deficiency observed in cancer cells, is impaired interferon-signaling. Interferons are important modulators of immune response, and defects in interferon signaling are highly detrimental to immune control of cancer cells [103]. Cytokine secretion is critically modulated by JAK/STAT activity [104].

#### Module 4

Finally, module 4 (Figure 6D) consists of the TAs EFTUD2 and SNRPD1 (Figure 5D), and their ten closest interaction partners. Seven of these proteins overlap in the spliceosome pathway, which plays a central role in pre-mRNA processing and splicing. Splicing in tightly regulated in different tissues and different stages of development, and dysregulation of the spliceosome function may lead to incorrect assembly of exons and nonfunctional translation. As such, alternative splicing plays a significant role in cancer and other malignancies [105]. The spliceosome has therefore been examined for its potential as a therapy target [106]. Spliceosome-related therapy can be directed towards the products of alternative splicing [107] or the spliceosome modulators [108].

### Non-interacting TAs

In addition to the four modules, we also observed 9 TAs that do not connect with any other TAs (Figure 5C). These are not necessarily less valuable as targets, but the analyses performed here are much less comprehensive for these TAs. Their interactants and involvement in molecular pathways relating to cancer is summarized in Additional file 1.

## Conclusions

Statistical testing for patterns in high throughput mRNA expression data has long been the primary method for defining biomarkers in human cancers. The analyses are constantly refined with inclusion of data from epigenetic experimentation, measurements of ncRNAs, and protein expression, and are expanded with ontology enrichment analyses, pathway analyses, and co-analyses of different data types. Additionally, as experimental methods increase in efficiency and resolution, the bodies of data examined keep growing. A large number of diagnostic and prognostic biomarkers have been reported, and a small number are utilized in clinics, but it is believed by some that the majority of reported biomarker candidates are the result of stochastic noise within data sets [109].

TAs are a group of proteins against which the immune system has been recorded to autologously react. Specifically, they are recognized in cells where they are present in larger than usual amounts, or physiochemically altered to a degree at which they no longer resemble native human proteins. As such, their presence or abundance in cancer cells is often unique and their roles and functions are, in many cases, studied extensively. Proteins that are frequently observed (and autologously recognized by the immune system) in tumor cells can be hypothesized to play a significant role in tumorigenesis. They therefore hold the potential to be highly specific biomarkers for the cancers in which they are observed.

The challenges pertaining to the utility of TA biomarkers are similar to those we face when we statistically filter out potential biomarkers from vast amounts of high throughput genomics data: our understanding of their function and role in cancer must be elevated to a degree where we can account for outliers and exceptions to the general rules known from clinical observations. To achieve this, we analyzed the mRNA and protein expression of 30 TAs in normal tissue versus IDC tissue. We found that all but two TAs were expressed in IDC on the protein level, and a subset of these was aberrantly expressed on mRNA level. We examined their known and proposed roles in cancer by analyzing the TAs and their closest functional counterparts for overlapping participation in canonical pathways. With this approach, we defined four functional modules of TAs and interactants, which overlapped in canonical pathways. The perturbation of these pathways were readily linked to the hallmarks of cancer by querying relevant literature. A previous study of genetic biomarkers in IDC tissue resulted in the identification of approximately 2,000 differentially expressed genes involved in a large number of biological pathways [37]. Among these pathways were those related to the hallmarks of cancer that we have found from analyzing only 30 TA genes.

Currently, 258 TAs are catalogued and annotated in the TANTIGEN database of TAs. Expanding the analysis performed here to the full set of TAs is highly likely to provide additional insights. RNA sequencing is another desirable follow-up study of TAs found to be expressed in a given cancer tissue, as this may reveal known or novel splice isoforms, mutations, or other genetic aberrations. In addition to the diagnostic and prognostic potential of such a study, a catalogue of expressed TAs and variants in a given tumor can be further analyzed for their potential as therapeutic targets and directly applied in personalized treatment modalities.

## Methods

### Data

#### Protein expression data

Nine samples of estrogen receptor positive ER+ IDC tissue were analyzed for expression of 1623 proteins using liquid chromatography coupled with mass spectrometry [43]. The tissue samples were obtained using laser capture microdissection from biopsies collected at the Massachusetts General Hospital. In addition to the nine IDC samples, nine (non-paired) normal tissue samples were analyzed for protein expression.

#### mRNA expression data

The mRNA expression data were extracted from breast cancer biopsies, again collected at the Massachusetts General Hospital [37]. Three different types of samples were collected: normal tissue, preinvasive tissue, and invasive tissue of ER+ IDC. The tissue consisted of cells from the epithelial and stromal compartments of the normal terminal ductal lobular unit. The following samples were paired: nine samples of normal and preinvasive cancer, nine samples of normal and invasive tissue, and four samples for all three tissue types. Tissue samples were obtained using laser capture microdissection and were analyzed for mRNA expression using the Affymetrix whole genome array U133X3P [38].

#### Tumor antigen data

A list of known TAs was extracted from the TANTIGEN database of TAs http://cvc.dfci.harvard.edu/tadb/. TANTIGEN contains 4245 T cell epitopes found in 258 unique protein TAs (November 2013) collected from the literature. Protein expression was measured for 32 of these TAs in [45]. Of these, gene expression was meassured for 30 TAs [38]. We further analyzed these 30 TAs by proteogenomics.

#### mRNA and protein expression in IDC tissue

For the analysis of correlation between gene expression and protein expression, we examined 404 IDC tissue samples collected by The Cancer Genome Atlas (TCGA) consortium. Paired mRNA expression and protein expression data was available for 86 gene/protein pairs, of which 13 are known TAs. mRNA expression was extracted using Agilent mRNA expression microarrays, and protein expression was extracted using Reverse Phase Protein Arrays [63].

### Analyses

#### Protein expression analysis

The TAs were extracted from the protein expression data. The nine normal samples were averaged and compared with expression in the nine invasive tissue samples. Log_2 _ratios of expression of each protein compared with the normal tissue average (normalized to 1 if expressed, or kept at 0 if not expressed) were calculated for each patient.

#### mRNA expression analysis

Raw probe intensities were background corrected using rma, quantile normalized [110,111], and the probe sets were indexed relative to the median. Fold changes were calculated as individual mRNA expression compared to the median mRNA expression and log_2 _transformed.

TA genes were extracted from the expression data. Fold changes were calculated between the three types of cancer tissue (normal, preinvasive, and invasive). Genes were examined for consistently differentially expressed genes in the patient cohort using a paired t test. Additionally, gene expression dynamics in the three cancer tissue types were examined in each individual patient.

#### Annotation

Each TA was characterized for its potential functions in tumorigenesis in each patient to gain an insight into the underlying biology of the observed expression profiles. Information about protein function and role in disease were extracted from OMIM [112], UniProt [113], GeneCards [114], the Human Protein Atlas [115] and UniGene http://www.ncbi.nlm.nih.gov/unigene.

#### Pathway analysis

Functional neighbors to aberrantly regulated TAs were extracted from the STRING database of protein-protein interactions (version 9.05) [116]. Interactions with > 0.5 confidence score were considered and the resulting protein groups were analyzed for their overlapping involvement in canonical pathways using the molecular signatures database, MSigDB [87]. The confidence score in STRING database is calculated from combined and corrected probabilities using different evidence channels for protein-protein interaction, described in [117].

#### Correlating mRNA and protein expression

To determine correlation between mRNA expression and protein expression, we calculated Spearman's rank correlation coefficient. The threshold for correlation was set at ρ > 0.455 to compare results with a previous study of correlation between the mRNA and protein expression [62]. The authors used the ρ > 0.455 threshold by determining that the average correlation coefficient of expression in 1000 randomly chosen gene/product pairs from 23 cancer cell lines was 0.001, and that the selected value of ρ of 0.455 provided the 95% confidence interval of the ρ distribution.

## Competing interests

The authors declare that they have no competing interests.

## Authors' contributions

LRO: Conceived of the study, performed the study, and prepared the manuscript.

BC: Contributed to the study of mRNA/protein pairs and critically reviewed manuscript.

OW: Contributed to the study of protein-protein interaction and critically reviewed manuscript.

DCS: Contributed to the study of gene expression and critically reviewed manuscript.

BLK: Contributed to the study of protein expression and critically reviewed manuscript.

VB: Conceived of the study and prepared the manuscript

## Supplementary Material

Additional File 1 TA, their interactants and involvement in molecular pathways relating to cancer. Click here for file
